# Managing chest pain patients in general practice: an interview-based study

**DOI:** 10.1186/s12875-018-0771-0

**Published:** 2018-06-02

**Authors:** Leen Biesemans, Lotte E. Cleef, Robert T. A. Willemsen, Beatrijs B. N. Hoorweg, Walter S. Renier, Frank Buntinx, Jan F. C. Glatz, Geert-Jan Dinant

**Affiliations:** 1 Department of Family Medicine, Catholic University Leuven, Leuven, Belgium; 2 Department of Family Medicine, Maastricht University, P. Debyeplein 1, (PO box 616), Maastricht, 6200 MD the Netherlands; 3 Department of Genetics & Cell Biology, Maastricht University, Maastricht, the Netherlands

**Keywords:** General practice, Cardiovascular disorders, Diagnostic tests, Urgent care, Risk assessment

## Abstract

**Background:**

Assessment of chest pain in general practice is challenging. General practitioners (GPs) often feel uncertainty when dealing with chest pain. The role of new diagnostic tools is yet unclear.

Therefore, we aimed to learn: (1) whether or not GPs experience a change in incidence and presentation of chest pain, (2) how GPs deal with uncertainty, and (3) which thoughts, demands and doubts concerning new diagnostic tools occur.

**Methods:**

Semi-structured, face to face interview based study, aiming at six main subjects: experienced changes in prevalence of chest pain, the management of chest pain patients, dealing with uncertainty, the GPs’ approach in referring chest pain patients, GPs’ attitude towards ‘unnecessary’ referrals, and the GPs’ suggestions for improving the management of chest pain patients.

**Results:**

145 GPs in Belgium and the Netherlands were invited to participate, 27 (15 Flemish and 12 Dutch) GPs were interviewed. Data saturation was reached. The number of patients having an acute coronary syndrome among chest pain patients is decreasing, whereas the presentation of atypical complaints increases, together leading to more uncertainty. GPs rely on their own judgment above all, and desire new diagnostic tools only when these tools are of proven added value.

**Conclusion:**

The incidence of chest pain in general practice is not decreasing according to the GPs. However, the presentation of chest pain is changing. GPs feel relatively comfortable with referring a considerable number of chest pain patients without ACS, as over-referral is safe. Uncertainty is regarded as a substantial element of their profession. New diagnostic tools are awaited with cautiousness.

**Electronic supplementary material:**

The online version of this article (10.1186/s12875-018-0771-0) contains supplementary material, which is available to authorized users.

## Background

### Chest pain in general practice

In general practice, chest pain as a reason for encounter is common (prevalence 1–3%) and differential diagnosis is broad [1, 2]. Yet, in only a minority, an acute life threatening disease is concerned. Severe diseases as acute coronary syndrome (ACS), pulmonary embolism or thoracic aortal dissection are outnumbered by non-urgent causes as gastro-esophageal reflux disease (GERD) or thoracic wall pain [1, 3, 4]. However, discriminating between ACS and less severe causes of chest pain, based on clinical findings or electrocardiography, is difficult [5–8].

### The challenge of (not) referring patients with chest pain

Most guidelines clearly state that general practitioners (GPs) should refer every patient suspected of an ACS to secondary care facilities as soon as possible, or GPs should even be bypassed to prevent loss of time and, consequently, myocardial cell necrosis [9]. However, for every chest pain patient with a life threatening disease as ACS, a GP encounters 11 patients with chest pain of a non severe cause [1]. Therefore, clinical judgement and triage by GPs remains inevitable to prevent unnecessary referrals and to keep the burden on secondary care facilities acceptable [3, 10]. To reach this goal, GPs compromise between adequate detection of severe disease on the one hand and reassurement in cases of worried patients with no or mild underlying disease on the other hand [6, 11, 12]. Over time, GPs seem to have found an optimum. They refer only a minority of chest pain patients, thereby adequately maintaining their gatekeeping role. Still, a considerable number of patients with chest pain (approximately 20–40%) are referred for safe exclusion of ACS, whereas only one out of five of these referred patients suffer from severe disease [1, 13, 14]. Besides classical clinical findings, GPs use gut feeling and background knowledge of their patients to make these challenging referral decisions [15].

### Uncertainty when dealing with chest pain

Notwithstanding this strategy to work with low thresholds for referral to maintain the number of missed cases of ACS low, uncertainty among GPs has been a part of dealing with chest pain in general practice for decades [16–18]. GPs regard guidelines as only partially solving their dilemmas in assessing chest pain patients and as suboptimally answering their questions whether or not to refer these patients [16]. As a possible consequence, guidelines do not integrally lead to behavioural change [19]. Besides, referring patients without consultation – for example based on a typical pattern presented in a phone call, as is advertised in cases of suspected stroke – is impracticable in cases of chest pain due to the broad differential diagnosis [20]. Cautious GPs that are anxious about the consequences of missing severe disease refer more often to secondary care facilities, especially when working in out of office hours services [21, 22]. Especially during consultations during out of office hours, maintaining a threshold for referral is challenging [17].

### Developments in the field of chest pain in general practice

Several developments in the field of chest pain in general practice have been described. First, an altering presentation of ACS in general practice has been reported, whether or not due to sex differences in presentation [5, 23–25]. Second, incidence of ACS among chest pain patients in general practice seems to be declining over the years (from approximately 20% to less than 10%), mortality has decreased strongly, and prognosis has improved [1, 26, 27]. Besides absolute data on this topic addressed in quantitative studies, a GP’s perception of a possible changing incidence of ACS is important, since a GP’s estimation of the probability of an ACS when assessing patients presenting with chest pain (the ‘pre-test probability’) is influenced by this perception [28, 29]. Third, several diagnostic studies have recently evaluated the role of new diagnostic tools – e.g. clinical decision rules (CDRs) and point-of-care tests (PoCTs) – in managing chest pain patients. GPs in Europe desire a PoCT especially in the field of patients presenting with chest pain [30]. However, a CDR safely reducing referrals is lacking [31].

### Objectives

Studies on GPs’ contemporary perceptions, attitudes and experiences in dealing with chest pain patients regarding uncertainty and developments in presentation, incidence and diagnostic tools as described above are mainly dealing with specific diseases, are focussing on out of office hours working situations or are lacking [16, 17, 32]. Therefore, in our study, we aimed to explore the following topics:

1. Do GPs experience a changing incidence and presentation of chest pain and / or ACS in daily practice, and if so, what changes do GPs observe?
2. Which considerations are important to GPs and how do they deal with uncertainty (regarding referrals of patients without a life threatening disease as well as missed cases of severe disease) in the management of chest pain patients?
3. Which thoughts, demands and doubts concerning new diagnostic tools can be extracted from GPs’ reflections on managing patients with chest pain? (This final question was addressed only in the Dutch interviews, since most Flemish GPs have no experience with such tools).

## Methods

### Inclusion

145 GPs were selected from a list of GPs who had indicated earlier they were interested in participating in scientific studies. We invited 15 Flemish GPs by phone and 30 per e-mail. 100 Dutch GPs were invited per e-mail and were phoned shortly afterwards to inquire possible participation. We included general practitioners willing to participate, with at least 5 years of working experience. No further selection criteria were used.

### Interview

The semi-structured face-to-face interview, composed by the Flemish researchers, consisted of five main subjects to answer the objectives of the study: (1) recently experienced changes in the prevalence of chest pain and ACS, (2) management of chest pain patients in daily practice, (3) dealing with uncertainty, (4) the GPs’ approach in referring chest pain patients, and (5) their attitude towards - in the light of the final diagnosis - ‘unnecessary’ referrals. The second subject (2) was approached by letting the GPs imagine a chest pain patient and thereby reflect on their diagnostic steps and decision to refer. An additional subject was added in the Dutch interview: (6) the GP’s own suggestions for improving the management of chest pain patients in general practice. The interview protocol was piloted with a Flemish and a Dutch GP who both had over 25 years of working experience. Flaws were discussed by the researchers and two GPs, which resulted in an adapted version of the interview. The pilot interviews were not included in the final research sample. The main questions, used as starting points of different phases of the interview are given in Additional file 1. Additional questions were not predefined, further answers and themes were initiated by the GPs themselves. The interviews took place at the GPs’ practices, incidentally in a GP’s private home.

### Data collection

Data were collected through face-to-face interviews. Flemish interviews were carried out by LB, as part of her research thesis completing her GP education, between June and October, 2015. Dutch interviews were carried out by LC and BH, both fulfilling their research internships during the master phase of their medical school, between April and June, 2016. All interviews were audio-recorded and transcribed verbatim. Data were collected until saturation was achieved. Saturation was defined as the identification of no new nodes in the last two interviews in the Flemish, or the Dutch part of the study, respectively.

### Data analysis

Thematic analysis was used to bring forth answers to the main objectives of this study. All Flemish interview transcripts were analysed independently by two researchers in a process of inductive line-by-line coding. LB coded all transcripts, and four GP residents and one anesthesiology resident coded three transcripts each. Afterwards, differences were discussed and further analysis was assisted by FB and WR. Thereafter, the initial codebook was established, which was adjusted after every four interviews. In a second step the codes were refined, resulting in the development of descriptive themes. Finally, a thematic analysis was performed by all researchers to generate new analytical themes.

Each Dutch interview was analysed and coded independently using the Flemish codebook by LC and BH. Conflicts were solved by discussion. During the analysis of subsequent interviews, the initial code list was further refined by adding new codes under the pre-existing themes, when confronted with relevant data that could not be linked to an existing code. Finally, a framework analysis was performed by LC, BH, and RW to generate new hypotheses. Ultimately, Flemish and Dutch results were independently analysed and discussed by LB, LC and RW to uncover similarities and differences in outcomes. Subsequently, these findings were documented when applicable in the results section of this paper. NVivo 11 pro software was used to facilitate coding.

### Ethics

All participants were informed about the aims of the study and the recording of the interviews. The study was approved by the Medical Ethical Committees of the University of Leuven and of Maastricht University.

## Results

145 (45 Flemish, 100 Dutch) GPs were invited to participate, 27 GPs (15 Flemish and 12 Dutch) were interviewed. The main reason for declining our invitation was a lack of time. GP characteristics are presented in Table 1. During the interviews, all GPs commented extensively on the questions concerning this study’s main three objectives. All interviews lasted 45 to 60 min, incidentally an interview lasted 75 min.

**Table 1:** Characteristics (gender, age, years of experience, type and area of practice) of the participating GPs

		Dutch	Flemish	Total
Gender	Male	8	10	18
	Female	4	5	9
Age groups	35–39	1	2	3
	40–44	4	0	4
	45–49	0	4	4
	50–54	1	2	3
	55–59	5	2	7
	59–65	1	4	5
	> 65	0	1	1
Years of experience		20.6 (2.5–36)	26.7 (10–40)	24.0 (2.5–40) ^a^
Type of practice	Single	4	4	8
	Duo	5	4	9
	Group	3	7	10
Area of practice	Urban	8	6	14
	Rural	4	9	13

Through purposive sampling we aimed to attain a heterogeneous group, including both female and male GPs, GPs working in both rural and urban regions and in single, duo and group practices. One participating GP had only 2,5 years of experience. Yet the data derived from this interview were maintained, since these were in line with the data from the more experienced GPs. Single, duo, group practices refer to GP practices managed by respectively one, two and more GPs. Abbreviations: *GP* general practitioner, *N* number. ^a^ Mean followed by the range in brackets

### Incidence and presentation of chest pain in general practice (objective 1)

Most GPs stated that they are confronted with chest pain at least once every week. Chest pain can have a wide variety of causes, of which the majority is not of cardiac origin.

“Hm, I think we are confronted with this complaint weekly, maybe even multiple times a week.” [NL GP 12]

“Yes, the lion’s share here, of people presenting with chest pain, is not of cardiac origin.” [NL GP 8]

Although some Dutch GPs experienced an evident increase in the prevalence of chest pain in their practice, the majority of GPs did not experience a change in frequency over time. However, most GPs experienced a change in the clinical spectrum of chest pain. GPs claimed to encounter less cases of ACS, due to improved cardiovascular risk management in general practice, and more atypical thoracic complaints, due to a growing awareness on chest pain in the general population. The anxiety accompanying this awareness, combined with the continuously available health care in large out-of-hours general practice facilities, lower the threshold to consult a GP.

“Then I think it is more or less the same … if you don’t only count cardiac but also other types of chest pain like respiratory infections or indeed psychosomatic symptoms. Then I think it [the frequency of chest pain] is comparable to before.” [BE GP 4]

“I think that the frequency with which we see a grave acute infarction nowadays is a lot lower than it was twenty years ago.” [BE GP 7]

Most GPs believed raising awareness in the general population on the possible consequences of chest pain is important. However, Dutch GPs stated it can result in an increase in patient anxiety, leading to more uncertainty and consultation of a GP.

“Of course raising public awareness is helpful, but it can also cause more unnecessary consultations. Not everyone is good at assessing their own situation. And people are worried when they feel something.” [NL GP 12]

Especially in out-of-hours general practice facilities in the Netherlands, new triage guidelines with a low threshold for directly sending out an ambulance in case of chest pain, are causing Dutch GPs to encounter less chest pain patients. Some Flemish GPs stated that recently more patients go directly to the emergency room, especially in urban areas with a high number of immigrants.

“Outside office hours people often call, saying: “I experience an acute severe pressure on my chest”, and according to the current guidelines, the ambulance will leave for this patient. In such case we don’t assess the patient ourselves.” [NL GP 8]

“We have a varied patient population here. We are on the verge of a rural area where most people still obediently go to the GP. On the other hand, in the city of Mechelen e.g., the citizens – and certainly the immigrants – go to the emergency department much easier and will thus pass us by.” [BE GP6]

### Assessment of chest pain and dealing with uncertainty (objective 2)

When confronted with a chest pain patient, GPs quickly attempt to distinguish between acute and non-acute pathology. The most important tools in assessing chest pain are history taking, clinical evaluation and gut feeling. The language used by GPs suggests a feeling of responsibility.

“Yes, you first try to determine in which framework the pain fits. (…) Are the lungs causing the problems, or is it the heart or is it something less serious? So that’s what you try to assess first and foremost.” [BE GP10]

“But I taught myself, if I have a certain gut feeling that something isn’t right, yes, then I just refer them. I’m not going to take the risk behind the patient’s back.” [NL GP 11]

Most GPs questioned the value of diagnostic tools such as ECG or troponin in ruling out ACS at first assessment. However, a few GPs felt confident enough to not refer a patient based on a negative ECG. In non-acute situations, these diagnostic tools are mostly used to take away any doubt, experienced by the GP, and to reassure the patient. GPs regard personal judgment, not advanced diagnostic tools, as the main instrument to rely on.

“Especially because you have to assess the value of an ECG. I mean that an ECG can be negative despite the fact that the clinical presentation is very suspicious. Then you have to follow your clinical judgment. The technicalities can be an affirmation of your clinical assessment but not vice versa.” [BE GP8]

“It also helps to reassure people that are not expected to have a serious condition, then you’ll have an additional confirmatory tool [ECG].” [NL GP 12].

When a patient is suspected of ACS, GPs immediately refer them to the emergency department, transported by ambulance with the highest level of urgency.

“But when someone has acute chest pain suspected of ACS, that goes with getting picked up by an ambulance with the highest urgency.” [NL GP 8].

Some GPs considered it acceptable for a patient to leave for the hospital on their own, if the patient is in a stable condition. However, they should never drive themselves.

“Or it is a semi-acute problem and the person can go to the hospital by their own means of transportation, transported by someone else. They shouldn’t drive themselves.” [NL GP 2]

Before the ambulance arrives, some GPs administer medication and accomplish intravenous access. However, a few Flemish GPs felt insecure about their knowledge on acute interventions and their necessity in urgent situations.

“Imagine if you had to suddenly give an injection urgently: (...) If you haven’t done any of that in twenty years, or haven’t received any information about it anymore, you won’t start doing it again that easily, right?” [BE GP10]

When patients call their GP because of chest pain and their situation seems to be unstable, it depends on the amount of time available, whether the GP heads to the patient himself, sends an ambulance directly, or does both.

“Unless I have to drive for fifteen minutes, some of our patients live quite far off, and I am really busy with other obligations. But in principle I do both [send an ambulance and leave for the patient].” [NL GP 2]

In non-urgent cases patients are referred to the hospital cardiology department for a consultation on a following day.

To GPs, the greatest difficulties in the assessment of chest pain are atypical symptoms, expressed – according to Dutch GPs – mainly by women.

“Because a woman often has atypical complaints regarding cardiovascular conditions. You have to be more careful, which means you maybe have to look a little closer.” [NL GP 7]

Some GPs feel the need to refer anxious patients, for their own and the patient’s peace of mind.

“I think when people keep consulting you with a certain complaint, not just chest pain, even if they have already consulted you about this complaint earlier, that’s an indication to refer the patient anyway.” [NL GP 2]

In urgent cases, most GPs feel no insecurity in choosing to refer a patient or not. However, when GPs experience uncertainty, it is mainly due to doubt as to which condition is causing the chest pain, sometimes leading to more referrals.

“But my rule is: when I’m uncertain, I always refer the patient.” [NL GP 10]

In less urgent cases some GPs try to perform most diagnostics themselves, start a test treatment, or opt for the approach of watchful waiting, to make a more targeted referral.

“Yes, I try to rule out some things myself, those things that are really easy to rule out. (…) And subsequently, because of these possibilities, you refer less often.” [NL GP 10]

To diminish uncertainty, GPs stated they could consult colleagues or a cardiologist. However, not all GPs feel the need to do so. Mainly Flemish GPs stated they rarely ask for advice on managing chest pain patients.

“I think that we are in a luxury position because we work here with three GPs in a group. There is always someone you can immediately ask for a second opinion. That helps a lot. A short discussion is often enough to be able to make a decision.” [BE GP5]

Sometimes, uncertainty remains after a consultation with a chest pain patient. To reduce this uncertainty, some GPs tend to refer more patients.

“Yes, if you have a feeling that you’ve made a mistake somewhere or that you’ve misjudged something it certainly keeps…lingering in your head for a while, keeps bothering you for a while.” [BE GP14]

Afterwards, some GPs like to debrief cases they were uncertain about, both on medical and personal aspects, either through the cardiologist’s letters or through discussion with colleagues. However, some Flemish GPs find it difficult to find a safe environment to talk about their uncertainty.

“It helps me that I can debrief about it … eh … in a safe environment. I think that that is one of the most important things a GP – and actually any doctor – can attain: safe situations. I’ve been working now for twenty years and I have finally found safety. (…) I think that that’s important for every doctor, that he has the opportunity to talk about his uncertainties with his peers.” [BE GP1]

Nonetheless, all GPs believed that insecurity and mistakes are part of their profession and that GPs must learn to handle these situations. Most GPs believed they became more confident in assessing chest pain during their career, due to experience and development of their gut feeling, causing them to refer less patients unnecessarily. Still, GPs stated they refer quite a large part of chest pain patients because they do not want to risk missing ACS. Looking back after obtaining the final diagnosis, some of these referrals were unnecessary. However, GPs believed this is due to a lack of diagnostic tools to initially rule out ACS. Critical incidents of missing ACS cause GPs to be more cautious, either temporarily or permanently. GPs judge their referral decisions as right or wrong in the light of a final diagnosis. This is remarkable, since a referral decision based on considering a severe disease can essentially not be wrong.

“I think the experience and the development of the gut feeling plays a role in becoming more confident. So, I think that I was more insecure 15 years ago, and because of that I may have referred more patients wrongly.” [NL GP 2]

“It is like that, when you get a claim or you think: “Oops, did I miss that?” then you will pay more attention to those complaints for a period of time. Absolutely.” [NL GP 5]

“Sometimes you have to make a risk assessment. Sometimes you are right and sometimes you are wrong. But there’s no dishonour in that. I would rather refer one patient too many than one too few. Because then you are going to have to come up with a really good explanation.” [BE GP3]

Referrals of patients that eventually appear to have no severe disease can be unfavourable, leading to high health care costs, an overload of the hospital staff and a stressful experience for patients. However, some GPs never let their referral decision depend on such possible thresholds. Others stated they try to prevent medicalisation and somatisation in low-risk patients. GPs sometimes get criticised on their referral decisions by hospital physicians. Most GPs claim not to be affected by this criticism, however, their language to express their feeling of making autonomous referral decisions is rather explicit. Thus, GPs seem to define their position in a difficult field where colleagues and patients might judge the GP’s decisions.

“I have assessed the patient, so I have to take responsibility and I have to make the decision on my own.” [NL GP 1]

“That is your autonomous decision. He wasn’t there at the moment you called. And if you say in that moment ‘I want an emergency doctor here’, then it has to be there, end of story! Ten minutes later, the situation is different, the insight is different, the patient is different and so on. And then maybe the decision would be different. But they can never judge you for that!” [BE GP 1]

### Thoughts, demands, and doubts concerning new diagnostic tools (objective 3)

Most GPs stated they are satisfied with the current diagnostic options for chest pain patients in general practice and some believe that clinical assessment will remain the most important tool in assessing chest pain patients. Others do believe an additional diagnostic tool such as a biomarker PoCT would be useful. Yet, usability strongly depends on indications and diagnostic accuracy. A few Dutch GPs suggested to combine such test with a clinical decision rule. In general, new diagnostic tools are considered to be of a certain degree of added value, with a remaining central role for clinical judgment by the GP.

“Look, if you just… Independently of what it says, and I think the patient’s situation is not looking good based on the clinical assessment, then I’ll refer him anyway. Then I don’t let it depend on a biomarker.” [NL GP 12]

“It [PoCT] might contribute something in case of doubt. Then of course it also depends on the sensitivity and the cost price. You have to be quite sure when you use such a thing and it comes out positive, that you won’t make big effort which in the end turns out to be futile.” [BE GP8]

“If a clinical decision rule will be designed (...) those are really helpful. You count the points and then you get yes, no, or an intermediate. Yes, if you add a troponin test to that (...) then I think it will contribute of the quality of health care.” [NL GP 8]

## Discussion

### Summary of main findings

First, when suspecting an ACS, GPs base their suspicion mainly on history taking and gut feeling. Second, recent findings in literature are endorsed by the interviewed GPs: chest pain is still a common reason for consulting a GP, the relative number of ACS patients among chest pain patients seems decreasing, whereas the presentation of atypical complaints increases, leading to more uncertainty. Third, GPs compensate for the experienced feeling of uncertainty by referring patients, performing additional tests or discussing cases with colleagues. Besides, GPs regard uncertainty as a substantial part of their profession and they feel more certainty when working experience increases. Still, some GPs use explicit language to express their certainty, possibly reflecting an ongoing struggle to keep patients and colleagues in secondary care satisfied. Overall, GPs feel relatively comfortable with a certain degree of over-referral of chest pain patients, as over-referral is regarded as a safe strategy in dealing with a potentially life threatening condition. An ongoing and substantial role for clinical judgment by the GP is expected. Fourth, new diagnostic tools in general practice are anticipated with cautiousness. Proper clinical embedding of such tools is obligatory according to the interviewed GPs.

### Strenghts and limitations of the study

Before interviewing the GPs, an extensive tryout of the interview with an experienced GP, observed by another experienced GP, was carried out. Two pilot interviews were performed. All Flemish interviews were carried out by LB, a GP in training. Her own experience from working in general practice might have had an impact on the interviews. There might have been bias due to her own opinion on subjects that were discussed. On the other hand, her own learning process as a GP might have lead to a curious attitude. The Dutch interviews were carried out by LC and BH, both master’s students in medicine. They had no experience in managing chest pain patients themselves, possibly leading to a certain degree of open-mindedness although the lack of experience could have prevented them from thinking of all possible in-depth questions.

The population of interviewed GPs contained a good variety in area, type of practice and age (Table 1). They might represent a selection of GPs more than averagely interested in chest pain, although we did not find any evidence for such selection bias. Moreover, a selection of interested GPs would lead to an underestimation of experienced uncertainty, rather than an overestimation. Our study population consisted of more men (*n* = 18) than women (*n* = 9). However, differences of the answers between sexes or years of experience as a GP were not observed. All participating GPs seemed motivated to respond extensively to our questions. Theoretical data saturation was reached in the Flemish and Dutch interviews separately.

### Further findings and comparison to existing literature

The incidence of chest pain in general practice has not changed remarkably. However, most GPs do experience a change in the clinical spectrum of chest pain, encountering less cases of ACS, and in its presentation. These findings are in line with various epidemiological studies, although data of these studies were partially obtained in a hospital setting [26, 27]. The GPs attribute these changes primarily to better prevention and treatment strategies. Additionally, GPs find that there are more atypical presentations of chest pain, due to the growing awareness of the possible causes of chest pain in the general population. This causes chest pain patients to worry and consult their GP more often. Dutch GPs state that the prevalence of atypical chest pain in women has increased over the years, possibly due to the growing awareness on this subject, and find the management of these patients challenging. Women indeed present with atypical chest pain more often than men, but more research on the possibly different pathophysiology in women and men is needed, in order to reveal the relevance of the differences in presentation [25]. Both Dutch and Flemish GPs reported that, in recent years, more patients go directly to the emergency room.

GPs’ judgment of chest pain is based mainly on history, physical examination and gut feeling. Indeed, it is known that GPs use additional tools for assessment, such as background knowledge about patients and gut feeling [15, 33]. Most GPs question the value and applicability of currently available diagnostic tools such as ECG and troponin in the assessment of chest pain. However, some GPs do use them to take away doubt or to reassure the patient. When there is a strong suspicion of ACS, GPs immediately refer the patient to the emergency department. Although most GPs agreed that transportation by ambulance with the highest level of urgency is appropriate, some GPs send stable patients to the hospital by their own means of transport, which is debatable [34]. Before the ambulance arrives, GPs sometimes administer medication and accomplish intravenous access. However, several Flemish GPs are reluctant to do this, doubting their ability to perform these interventions. Studies on this subject are scarce. A cross-sectional study from 2008 showed that a training program for GPs on interventions for ACS could improve the pre-hospitalization care of these patients [35].

GPs sometimes experience uncertainty in the management of chest pain patients, during or after the consultation. GPs tend to reduce this uncertainty by easily referring patients or performing additional tests. Some GPs consult their colleagues or a cardiologist to diminish uncertainty, though mainly Flemish GPs rarely ask for advice on managing chest pain. When uncertainty persists after the consultation, most GPs seek confirmation either through the cardiologist’s letters or through discussion with colleagues. GPs overall become more confident in assessing and managing chest pain patients during their career.

GPs mainly feel comfortable with their diagnostic assessment, management and possible over-referral of chest pain patients, stating that sometimes referral is necessary to get confirmation of the diagnosis. For most GPs the unfavourable consequences of ‘unnecessary’ referrals and criticism by secondary care physicians are not influencing their decisions. Several studies indeed indicate that GPs’ clinical judgment is quite accurate and that they succeed in managing chest pain patients well [1, 13, 36]. However, the explicit language used when describing their feeling of certainty suggests that GPs are aware of underlying phenomena. These phenomena might be reflections of their strong feeling of responsibility when dealing with possibly life threatening disease and / or might be a consequence of the feeling of being judged by patients or colleagues for correctly referring.

Though most GPs agree that clinical assessment will remain the most important tool in diagnosing chest pain patients, some GPs think that reliable new diagnostic tools (such as PoCTs) could be a useful addition to improve diagnostic accuracy. The general attitude towards such tools seemed more conservative than in recent questionnaire based studies [30, 37]. GPs pointed out that such tools should be reliably embedded in clinical care and is regarded as an additional tool rather than an alternative one making clinical judgments unneeded in the future. Moreover, dilemmas in assessing chest pain patients are thought to partially persist, regardless of future developments.

## Conclusions

- GPs feel that the incidence of chest pain in general practice is not decreasing. However, the number of patients having an acute coronary syndrome among patients presenting with chest pain is decreasing, whereas the presentation of atypical complaints increases, together leading to more uncertainty. Yet, uncertainty is regarded as a substantial element of their profession.
- GPs feel relatively comfortable with referring a considerable number of chest pain patients without ACS, as over-referral is safe.
- New diagnostic tools are awaited with cautiousness: GPs rely on their own judgment, and desire new diagnostic tools only when these tools are of clear added value.

## Additional file

Additional file 1: Semi-structured interview. (DOCX 27 kb)

## Abbreviations

ACS: Acute Coronary Syndrome
CDR: Clinical decision rule
GERD: Gastro-esophageal reflux disease
GP: General practitioner
PoCT: Point of care test
