# Supplementary material for: Managing chest pain patients in general practice: an interview-based study
Source: BMC Fam Pract. 2018 Jun 2;19:80. doi: 10.1186/s12875-018-0771-0 (PMC5985055; doi:10.1186/s12875-018-0771-0)
Supplement: Supplementary file 1 — Semi-structured interview. (DOCX 27 kb) [file 12875_2018_771_MOESM1_ESM.docx]

**ADDITIONAL FILE 1**

**SEMI-STRUCTURED INTERVIEW**

**STEPS BEFORE START INTERVIEW:**

- Introduction of interviewer(s)
- Explanation of procedure, purpose, anonymous analysis, recording of interview.
- lnformed consent procedure.
- Explain: explorative interview, no right or wrong answers, chest pain is broader than ACS alone.
- Register GP's age, sex, region, practice type, years of experience, specific areas of interest, post academic education

**INTERVIEW:**

*A Epidemiology*

| **Main question 1:**   - **The interview is about chest pain of all kinds. Have you got an idea how frequently you are confronted with chest pain in your practice? Does the frequency of chest pain seem altered lately?** |
| --- |

*B Imagine a chest pain patient...*

| **Main question 2:**  **Imagine you're assessing a chest pain patient at this very moment. What factors determine whether or not you decide to refer the patient?** |
| --- |

*C Uncertainty*

| **Main question 3:**  **The differential diagnosis of chest pain is broad, some of the possible causes demand early recognition and action, e.g. ACS. It is imaginable that GPs are in doubt when faced with pain about their diagnosis and actions to take. How are your experiences on this topic?** |
| --- |

*D Actions*

| **Main question 4:**  **If you decide to refer a patients with chest pain, which further actions do you take?** |
| --- |

| **Main question 5:**  **We know from studies that GPs tend to refer a fair number of patients that turn out to not suffer from any serious conditions, in order to miss only few of such severe diseases. However, this leads to a high number of referral of patients not suffering from severe conditions? How do you see this?** |
| --- |

*E Future tools*

| **Main question 6:**  **How, to your opinion, can diagnostic assessment of chest pain patients in primary care be improved in the future?** |
| --- |

After the main interview: We have reached the end of the main interview. Have you got any questions, remarks or additions concerning the interview?

Whenever you have any questions or requests, please feel free to contact us..

Have you got any tips for us?

We will send you a copy of out paper once we have completed it.

Thank you very much for time and effort to participate in our study.

**Addendum A: the semi-structured interview used in the study**

*Six main questions were predefined as a starting point. Further questions were not predefined. The conversation on the six main topics was primarily lead by the GPs' answers. Additional questions were asked when appropriate. GPs were given free space to talk about other issues, whenever applicable.*

*Abbreviations: ACS = acute coronary syndrome ; GP = general practitioner.*
